# Conditions for the origin of homochirality in primordial catalytic reaction networks

**DOI:** 10.1038/s41598-023-36852-4

**Published:** 2023-06-19

**Authors:** Jean-Sébastien Gagnon, David Hochberg

**Affiliations:** 1 Physics Department, Norwich University, Northfield, VT USA; 2 Department of Molecular Evolution, Centro de Astrobiología (CSIC-INTA), Ctra. Ajalvir Km. 4, 28850 Torrejón de Ardóz, Madrid Spain

**Keywords:** Origin of life, Reaction kinetics and dynamics, Complex networks

## Abstract

We study the generation of homochirality in a general chemical model (based on the homogeneous, fully connected Smoluchowski aggregation-fragmentation model) that obeys thermodynamics and can be easily mapped onto known origin of life models (e.g. autocatalytic sets, hypercycles, etc.), with essential aspects of origin of life modeling taken into consideration. Using a combination of theoretical modeling and numerical simulations, we look for minimal conditions for which our general chemical model exhibits spontaneous mirror symmetry breaking. We show that our model spontaneously breaks mirror symmetry in various catalytic configurations that only involve a small number of catalyzed reactions and nothing else. Of particular importance is that mirror symmetry breaking occurs in our model without the need for single-step autocatalytis or mutual inhibition, which may be of relevance for prebiotic chemistry.

## Introduction

The empirical predisposition of biopolymers to be composed from homochiral L-amino acids and D-sugars towards a single handedness, or chirality, is a remarkable feature of terrestrial biochemistry and biology. Theoretical proposals in prebiotic chemistry suggest that this uniformity in the handedness of the chemical building blocks, or homochirality, emerged in nature in abiotic times through the action of deterministic or chance mechanisms^1–3^. The abiotic scenario for the emergence of single homochirality in the biological world implies that the asymmetry could have emerged provided a small chiral fluctuation with respect to the unstable racemic composition can be amplified to a state useful for biotic evolution. By virtue of this reasoning, experiments and theoretical and numerical modeling that demonstrate the feasibility of stochastic mirror symmetry breaking are particularly important^4–14^.

Stochastic or Spontaneous Mirror Symmetry Breaking (SMSB) can take place, by way of example, in chemical networks involving enantioselective autocatalysis, and when the reaction rate and flow rate parameters (in open reactors) take on certain values. In these situations, the deviation from the unstable racemic, or the mirror-symmetric, composition is not achieved by kinetic control but is instead a consequence of the existence of non-equilibrium stable and scalemic stationary states. This is so because, beyond a critical value of the entropy production, the racemic state becomes metastable along the thermodynamic branch and thus tiny compositional fluctuations about this branch allow the system to evolve to one of two energetically degenerate and equally probable chiral states. This dynamical phenomenon is described by a bifurcation scenario to final stable ordered states. In the organic chemistry parlance, this event corresponds to an absolute asymmetric synthesis (AAS) in the absence of any chiral polarization. Once the initial chiral fluctuation is generated by random chance events, the initial perturbation can then be transmitted to the remainder of the system provided that the symmetry breaking step is coupled to subsequent chemical transformations of efficient amplification, either by means of self-replication reactions, or by means of other nonlinear transformations. Relevant features that are common in all such systems are that they take into account the small fluctuations about the unstable mirror symmetric (racemic) state and they display nonlinear kinetic effects^15,16^.

Autocatalysis is usually considered to be a necessary condition for life^17–19^ and emerges during the evolutionary stage of the appearance of replicator molecules and template mechanisms of self-reproduction. The emergence of catalytic functionalities and autocatalytic systems are the basis of models for abiotic chemical evolution towards self-reproducing systems. Some of the well-known examples of this are given by autocatalytic sets^20^, quasi-species^21^ and the RNA world^22^. The coexistence, mutual stabilization and growth of self-reproducing chemical species is justified by models of sets of autocatalytic replicators^23,24^. The classic example is the Eigen-Schuster hypercycle model consisting of replicator sets coupled by mutual cross-catalysis^25^.

Uniform handedness or homochirality is ubiquitous in biological chemistry from its outset. In spite of this fact, there is a surprising and glaring omission of the chirality problem in many studies that are devoted to the abiotic stage of formation of the instructed polymers and replicators that could lead to the onset of Darwinian evolution (although see Refs.^26,27^ for examples where homochirality is considered in the context of the origin of life). The chiral structure and enantioselective kinetics in replicator models, autocatalytic sets, and of the RNA world are either completely passed over or else are simply and conveniently tacitly assumed. This means that the emergence of biological homochirality^2,28^ is somehow supposed to have taken place due to other processes separated from, or parallel to, the replicator formation. The emergence of biological homochirality is thus considered to be an accidental singular process of chemical evolution, and not the result of a collective emergent phenomenon linked to the increase of complexity in chemical evolution. Moreover, the formation of enantiomerically pure polymers is assumed to occur by starting from enantiomerically pure mixtures of their monomers. From the strictly chemical point of view, the latter supposition leads to an unlikely scenario because it implies the existence of pools of enantiomerically pure mixtures of amino acids and sugars. How should one justify such a macroscopic initial chiral purity? This scenario also does not take into account the fact that racemization is likely to occur, at least to some extent, over the tremendously long timescales of evolution and for the experimental conditions necessary for polymer condensation. It is an untenable assumption.

In this paper, we are interested in finding minimal conditions for homochirality to emerge in a general chemical model that can easily be mapped onto well-known origin-of-life models (e.g. autocatalytic sets, hypercycles, etc), with essential aspects of such modeling taken into consideration. We show here, how spontaneous mirror symmetry breaking can emerge from polymer growth dynamics when the chirality and the enantioselectivity of the oligomers is taken into account in tandem with their formation from monomeric chiral resources or food molecules. We consider a class of chiral catalytic reaction networks in an open flow continuous stirred-tank reactor (CSTR), which maintains the network out of thermodynamic equilibrium with its surroundings, that is, systems leading to non-thermodynamic final states as the most stable states of the system. This occurs in living biological systems^23^ and in systems leading to SMSB, rather than the racemic mirror-symmetric mixture^28^. In all our simulations, both the forward and reverse reactions are considered. This allows one to describe a system obeying the crucial constraints between the equilibrium constants and the reaction rate constants, as dictated by chemical thermodynamics^29,30^, and safeguards us against obtaining spurious SMSB results arising from overt violations of the second law of thermodynamics.

The remainder of this paper is organized as follows. We first present our chiral prebiotic inspired chemical model and discuss the relevant thermodynamic constraints on the dynamics. We then explore the parameter space of the model using numerical simulations, and discuss the implications in the Discussion.

## Presentation of the model

### Smoluchowski aggregation-fragmentation model

Our study of homochirality is based on the Smoluchowski aggregation-fragmentation model (e.g.^31–33^), modified by Giri and Jain^34^ to take into account catalysis. The model is based on the following (reversible) chemical reactions:

1 $$\begin{aligned} \textrm{C}_{i} + \textrm{C}_{j}&\mathop \rightleftharpoons \limits _{r_{ij}}^{k_{ij}}&\textrm{C}_{n} \end{aligned}$$

where $k_{ij}$ and $r_{ij}$ are symmetric matrices representing forward and reverse reaction rates, respectively. The subscripts on the C$_{i}$’s are used to label each molecule, and also serve to indicate its mass (in arbitrary units). Thus to conserve mass, we require that $i+j = n$ in Eq. (1). Note that in this notation, both $\textrm{C}_{i} + \textrm{C}_{j}$ and $\textrm{C}_{j} + \textrm{C}_{i}$ give C$_{n}$, implying that information about molecular structure is not taken into account in this model.

From mass action kinetics and assuming well-mixed conditions, we can obtain equations describing the time evolution of the concentrations of each molecule present in the system:

2 $$\begin{aligned} \frac{d C_{n}}{dt} \;=\; \sum _{i \le j, i+j=n} k_{ij} C_{i}C_{j} - C_{n}\sum _{i \le j, i+j=n} r_{ij} + \sum _{i = 1}^{N - n} r_{ni}\left( 1 + \delta _{ni} \right) C_{n+i} - C_{n}\sum _{i = 1}^{N-n} k_{ni}\left( 1 + \delta _{ni} \right) C_{i}, \; \end{aligned}$$

where the $C_{n}$’s now refer to the (time-varying) concentrations of the C$_{n}$ molecules. The first and fourth terms in Eq. 2 represent aggregation from smaller or to larger molecules (respectively), while the second and third terms represent fragmentation to smaller or from larger molecules (respectively). The sums in the second line are finite because of the maximum molecule mass in the system (denoted by *N*). The explicit Kronecker delta in the last two sums are there to take into account the stoichiometric factor of 2 arising in reactions of the type $\textrm{C}_{n} + \textrm{C}_{n} \rightleftharpoons \textrm{C}_{2n}$.

An important characteristic of living systems is that they possess a metabolism, where they extract energy and material present in their environment to operate and maintain their structural integrity. Said differently, living systems are intrinsically out-of-equilibrium. This can be accommodated in the Smoluchowski model by allowing it to be open, where fresh “food molecules” are fed in and products are taken out (keeping the volume constant). For each food molecule C$_{i}$ ($i = 1, \dots , M < N$), we add a constant inflow term of the type:

3 $$\begin{aligned} \frac{d C_{i}}{dt} &=  \left[ \frac{}{}\text{ reaction } \text{ terms }\frac{}{}\right] + \phi C_{0i}, \end{aligned}$$

where $\phi$ is the inflow rate (or the inverse residence time) of food molecules in the system and $C_{0i}$ is the input concentration of the $i\mathrm{th}$ food molecule. Note that food molecules are typically small (low mass) molecules that are used to build larger molecules. Thus for simplicity and in the spirit of searching for minimal conditions for homochirality, we only allow the smallest molecule in the system to be a food molecule (i.e. we take $M=1$ in the above). In addition, all molecules in the system are taken out at a constant rate $\phi$. This can be done by adding an outflow term to each molecule C$_{i}$ ($i = 1, \dots , N$) in the system:

4 $$\begin{aligned} \frac{d C_{i}}{dt}&=  \left[ \frac{}{}\text{ reaction } \text{ terms }\frac{}{}\right] - \phi C_{i}. \end{aligned}$$

Note that the outflow rate must be equal to the inflow rate in order to keep the volume constant. This type of modeling is in consonance with leading early-life scenarios such as hydrothermal fields, in which geothermally heated fluids flow into cavities present in submarine vents^35^ or surface pools^36^, and may lead to conditions conducive to the appearance of life.

Catalysis is also an important aspect of modern living systems, in which the rates of crucial chemical reactions are greatly amplified thanks to a plethora of efficient catalysts called enzymes. It is believed that catalysis (and in particular autocatalysis) also played an important role at the origin of life^17–19,25^. To include catalysis in the Smoluchowski model, one simply has to replace the reaction rates in Eq. (2) by the following:

5 $$\begin{aligned} k_{ij}\rightarrow & {} k_{ij}\left( 1 + \sum _{s} \kappa _{s}^{ij} C_{s}\right) , \end{aligned}$$

6 $$\begin{aligned} r_{ij}\rightarrow & {} r_{ij}\left( 1 + \sum _{s} \kappa _{s}^{ij} C_{s}\right) , \end{aligned}$$

where $\kappa _{s}^{ij}$ represents an enhancement factor (catalytic strength) due to the presence of a catalyst $C_{s}$, and the sum runs over all catalysts of the reaction. These catalysts are the same molecules appearing in Eq. (1), and are thus not external to the system. Note that the catalyst enhances the forward and reverse reactions with the same strength. Note also that this treatment of catalysis is simplistic, since it does not take into account the formation of intermediate complexes (for an explicit example of such treatment in the context of autocatalytic sets, see Refs.^37,38^).

Several comments can be made about this variant of the Smoluchowski model. First, the parameters $k_{ij}$ and $r_{ij}$ contain all the information about the chemical network. A value of zero implies that the two molecules do not react with each other, while a nonzero value implies some reaction. Thus nonzero values implies connectivity in the chemical network. For the purpose of this work, we define a fully connected chemistry as a network of chemical reactions that satisfies $k_{ij} \ne 0$, $r_{ij} \ne 0\; \forall i,j$.

Second, each matrix ($k_{ij}$ and $r_{ij}$) contain $\frac{N^{2} + 1}{2}$ parameters, but due to detailed balance, not all those parameters are independent of each other. These thermodynamic constraints are essential to prevent the appearance of perpetual motion machines in a chemical system that could lead to spurious mirror symmetry breaking^29,30^. They are discussed in detail below.

Third, the catalytic strengths $\kappa _{s}^{ij}$ are also parameters of the model that can be adjusted. The model contains sufficient freedom to allow the implementation of various catalytic configurations of interest to prebiotic chemistry modeling, such as self-catalysis, autocatalytic cycles^39^, autocatalytic sets^18,20^, and to a lesser extent, hypercycles^25^.

### Chiral Smoluchowski model

To study mirror symmetry breaking, it is necessary to “chiralize” the Smoluchowski model presented in the previous section. To do that, it is important to distinguish between left-handed (L) and right-handed (D) molecules (from the Latin words *laevus* and *dexter* for left and right), leading to two chiral sectors in which molecules of a certain handedness interact with molecules of the same handedness. Additionally, it is important to add a mechanism that allows interactions between the two chiral sectors. One such mechanism is enantiomerization^40^, in which molecules of a certain handedness turn into molecules of the opposite handedness through chemical interconversion (e.g.^41^) or tunneling (e.g.^42^), potentially leading to racemization. Assuming in the following that all molecules have a handedness (i.e. no achiral molecules present), a possible chiralized version of the Smoluchowski model is:

7 $$\begin{aligned} \textrm{C}_{i}^\mathrm{L} + \textrm{C}_{j}^\mathrm{L}&\mathop \rightleftharpoons \limits _{r_{ij}}^{k_{ij}}&\textrm{C}_{n}^\mathrm{L} \quad (i,j,n \le N,\; i+j=n) \end{aligned}$$

8 $$\begin{aligned} \textrm{C}_{i}^\mathrm{D} + \textrm{C}_{j}^\mathrm{D}&\mathop \rightleftharpoons \limits _{r_{ij}}^{k_{ij}}&\textrm{C}_{n}^\mathrm{D} \quad (i,j,n \le N,\; i+j=n) \end{aligned}$$

9 $$\begin{aligned} \textrm{C}_{i}^\mathrm{L}&\mathop \rightleftharpoons \limits _{f_{i}}^{f_{i}}&\textrm{C}_{i}^\mathrm{D} \quad (i \le M) \end{aligned}$$

10 $$\begin{aligned}&{\mathop {\longrightarrow }\limits ^{\phi C_{0i}}}&C_{i}^\mathrm{L,D} \quad (i \le M) \end{aligned}$$

11 $$\begin{aligned} C_{i}^\mathrm{L,D}&{\mathop {\longrightarrow }\limits ^{\phi }}&\quad (i \le N) \end{aligned}$$

where the superscripts (L,D) indicate the handedness of the molecule, *N* is the maximum molecule mass in the system, *M* is the maximum food molecule mass in the system (taken to be 1 in the following), and $f_{i}$ are the rate constants of enantiomerization (note that both forward and reverse rates must be equal, in order to not introduce any bias).

The time evolution equations for the concentrations corresponding to reactions (7)–(11) are:

12 $$\begin{aligned} \frac{d C_{1}^\mathrm{L,D}}{dt}&=  \sum _{i = 1}^{N - 1} r_{1i}\left( 1 + \delta _{1i}\right) C_{1+i}^\mathrm{L,D} - C_{1}^\mathrm{L,D}\sum _{i = 1}^{N-1} k_{1i}\left( 1 + \delta _{1i}\right) C_{i}^\mathrm{L,D} + f\left( C_{1}^\mathrm{D,L} - C_{1}^\mathrm{L,D}\right) + \phi \left( C_{01}^\mathrm{L,D} - C_{1}^\mathrm{L,D}\right) , \end{aligned}$$

13 $$\begin{aligned} \frac{d C_{n}^\mathrm{L,D}}{dt}&=  \sum _{i \le j, i+j=n} k_{ij} C_{i}^\mathrm{L,D}C_{j}^\mathrm{L,D} - C_{n}^\mathrm{L,D}\sum _{i \le j, i+j=n} r_{ij} + \sum _{i = 1}^{N - n} r_{ni}\left( 1 + \delta _{ni}\right) C_{n+i}^\mathrm{L,D} - C_{n}^\mathrm{L,D}\sum _{i = 1}^{N-n} k_{ni}\left( 1 + \delta _{ni}\right) C_{i}^\mathrm{L,D} - \phi C_{n}^\mathrm{L,D}, \;\;\;\;\;\;\; \end{aligned}$$

where we explicitly separated the equation for the food molecules ($$n=1)$$ from those for the non-food molecules ($$2 \le n \le N$$). Catalysis can be included easily using the replacements (5)–(6). Note that we assume the catalytic strengths for left-handed and right-handed molecules are the same (in order to not introduce any bias).

The nonlinear nature of Eqs. (12)–(13) makes them difficult to solve analytically, thus requiring the use of numerical methods. A systematic numerical study of the parameter space of the chiral Smoluchowski model would be extremely time consuming when *N* is large, due to the huge number of parameters involved. To make progress, it is necessary to make simplifying assumptions. The first one is that we consider a fully connected version of the chiral Smoluchowski model in the following. In addition, we consider a homogeneous chemistry, where all forward reaction rates are equal ($k_{ij} = k\; \forall i,j$), as well as all reverse reaction rates ($r_{ij} = r\; \forall i,j$). This last simplification drastically reduces the parameter space to be searched for mirror symmetry breaking, and can be partly justified in the context of isodesmic supramolecular polymerization^43^. Having isodesmic reactions is of course an approximation, as this would imply that molecules would become more stable as they increase in size, which is not true for polymers in general. Moreover, forcing all rate constants to be equal might seem to violate detailed balance, but we show in the next section that this choice is perfectly compatible with thermodynamics.

For numerical purposes, we make the chiral Smoluchowski model dimensionless by measuring concentrations in units of the input concentration of the food molecule $C_{01}^\mathrm{L}$ (or $C_{01}^\mathrm{D}$), since both must be equal in order to not introduce any bias), and measuring time in units of the inflow rate $\phi$:

14 $$\begin{aligned} \tilde{C}_{n}^\mathrm{L,R}&=  \frac{C_{n}^\mathrm{L,R}}{C_{01}^\mathrm{L}}, \end{aligned}$$

15 $$\begin{aligned} \tilde{t}&=  t\phi , \end{aligned}$$

where dimensionless quantities are represented with a tilde. Inserting the dimensionless quantities (14)–(15) into Eqs. (12)–(13), and applying the simplifying assumptions discussed above, we finally obtain:

16 $$\begin{aligned} \frac{d \tilde{C}_{1}^\mathrm{L,D}}{d\tilde{t}}&=  \sum _{i = 1}^{N - 1} \tilde{r}\left( 1 + \delta _{1i}\right) \tilde{C}_{1+i}^\mathrm{L,D} - \tilde{C}_{1}^\mathrm{L,D}\sum _{i = 1}^{N-1} \tilde{k}\left( 1 + \delta _{1i}\right) \tilde{C}_{i}^\mathrm{L,D} + \tilde{f}\left( \tilde{C}_{1}^\mathrm{D,L} - \tilde{C}_{1}^\mathrm{L,D}\right) + \left( 1 - \tilde{C}_{1}^\mathrm{L,D}\right) , \end{aligned}$$

17 $$\begin{aligned} \frac{d \tilde{C}_{n}^\mathrm{L,D}}{d\tilde{t}}&=  \sum _{i \le j, i+j=n} \tilde{k} \tilde{C}_{i}^\mathrm{L,D}\tilde{C}_{j}^\mathrm{L,D} - \tilde{C}_{n}^\mathrm{L,D}\sum _{i \le j, i+j=n} \tilde{r} + \sum _{i = 1}^{N - n} \tilde{r}\left( 1 + \delta _{ni}\right) \tilde{C}_{n+i}^\mathrm{L,D} - \tilde{C}_{n}^\mathrm{L,D}\sum _{i = 1}^{N-n} \tilde{k}\left( 1 + \delta _{ni}\right) \tilde{C}_{i}^\mathrm{L,D} - \tilde{C}_{n}^\mathrm{L,D}, \end{aligned}$$

with dimensionless rate constants given by:

18 $$\begin{aligned} \tilde{k}&=  \frac{C_{01}^\mathrm{L}k}{\phi }, \end{aligned}$$

19 $$\begin{aligned} \tilde{r}&=  \frac{r}{\phi }, \end{aligned}$$

20 $$\begin{aligned} \tilde{f}&=  \frac{f}{\phi }. \end{aligned}$$

To include catalysis, we make the replacements:

21 $$\begin{aligned} \tilde{k}_{ij}\rightarrow & {} \tilde{k}_{ij}\left( 1 + \sum _{s} \tilde{\kappa }_{s}^{ij} \tilde{C}_{s}^\mathrm{L,D}\right) , \end{aligned}$$

22 $$\begin{aligned} \tilde{r}_{ij}\rightarrow & {} \tilde{r}_{ij}\left( 1 + \sum _{s} \tilde{\kappa }_{s}^{ij} \tilde{C}_{s}^\mathrm{L,D}\right) , \end{aligned}$$

with dimensionless catalytic enhancement factors given by:

23 $$\begin{aligned} \tilde{\kappa }_{s}^{ij}&=  C_{01}^\mathrm{L}\kappa _{s}^{ij}. \end{aligned}$$

The fully connected, homogeneous, and dimensionless chiral Smoluchowski model defined by Eqs. (16)–(17) is the one we use to numerically study mirror symmetry breaking in the following. The model has only one food molecule ($M = 1$), and contains a total of four dimensionless parameters (*N*, $\tilde{k}$, $\tilde{r}$, $\tilde{f}$) in addition to a variable number of dimensionless catalytic enhancement factors ($\tilde{\kappa }_{s}^{ij}$). A summary of the relevant parameters for the model is given in Table 1.

**Table 1:** Summary of key parameters for the fully connected, homogeneous, dimensionless chiral Smoluchowski model.

Symbol	Definition	Reference in the text
N	Maximum molecule mass in the system	Below Eq. (2)
$\tilde{k}$	Dimensionless forward rate constant (same for all reactions)	Eq. (18)
$\tilde{r}$	Dimensionless reverse rate constants (same for all reactions)	Eq. (19)
$\tilde{f}$	Dimensionless enantiomerization rate (only for the smallest molecule in the system)	Eq. (20)
$\tilde{\kappa }_{s}^{ij}$	Dimensionless catalytic enhancement factors (same for all catalyzed reactions)	Eq. (23)

### Thermodynamic constraints

To study Eqs. (16)–(17) numerically, it is necessary to choose values for the various parameters contained in it. Care must be taken when choosing those parameters, since thermodynamics imposes important constraints that must be satisfied in order to have a meaningful chemistry without spurious behavior^29,30^. A chemical system in equilibrium must satisfy detailed balance (i.e. forward and reverse rates of all reactions must be equal), implying that forward and reverse reaction rates cannot be chosen arbitrarily for all reactions. Violating detailed balance would be equivalent to allowing for perpetual motion machines in the chemical system. As discussed extensively in the literature^44–47^, chemical systems driven out-of-equilibrium by some mechanism must also obey those thermodynamic constraints, in order for them to reach thermodynamic equilibrium once the driving mechanism is shut off.

A general treatment of thermodynamic constraints in chemical networks can be found in Refs.^48,49^. We review the general formalism below, and apply it to the fully connected homogeneous Smoluchowski model afterward. We start with a few definitions:

- *Number of reaction pairs* (p) Number of reaction pairs in a chemical network, equal to the number of reversible reaction arrows ($\rightleftharpoons$) in Eq. (1).
- *Number of complexes* (c) Number of distinct quantities that appear at the start or end of a reversible reaction arrow ($\rightleftharpoons$).
- *Number of linkage classes* (l) Also called number of components in graph theory, it is the number of connected subgraphs in a network that are not connected to the rest of the network. The components of any network partition its vertices in disjoint sets.
- *Spanning forest* In graph theory, a spanning tree of an undirected connected graph is a subgraph that is a tree which includes all of the vertices of the graph, where a tree is a an undirected graph in which any two vertices are connected by exactly one path (i.e. a tree cannot contain any cycles). Note that a graph may have many spanning trees (i.e. a spanning tree is not unique). When a graph is not fully connected (i.e. the graph has many components), then a spanning tree can be obtained for each component separately. The union of these spanning trees form a spanning forest.
- *Number of fundamental cycles* ($\gamma$) This quantity gives the number of fundamental cycles in the chemical reaction network. By cycle we mean a connected reversible subgraph in which each complex is directly linked by reversible reaction pairs to precisely two other complexes (i.e. a loop that starts from one complex and ends at the same complex, with the symbol $\rightleftharpoons$ linking each complex). It can be shown that $\gamma = p - (c-l)$.
- *Deficiency* ($\delta$) The deficiency of a chemical network can be computed from the formula $\delta = c - l - s$, where *s* is the rank of the stoichiometric matrix of the network.

With this terminology defined, we can state a theorem due to Feinberg^48^:

#### Theorem 1

Consider a mass action system in which the underlying reaction network is reversible, has a defiency of $\delta$ and, moreover, has *p* reaction pairs, *c* complexes and *l* linkage classes. Consider also a fixed (but arbitrary) choice of a spanning forest for the network. The system is detailed balanced if and only if the rate constants satisfy $\gamma$ cycle conditions and $\delta$ spanning forest conditions.

The usefulness of the above theorem lies in the fact that it gives necessary and sufficient conditions on the rate constants to obtain detailed balancing. By computing $\gamma$ and $\delta$ for a given chemical network, we know exactly how many constraints on the rate constants are needed to satisfy detailed balance.

As stated in the theorem, there exists two types of detailed balance conditions: cycle conditions and spanning forests conditions. We show below that $\gamma =0$ for the fully connected Smoluchowski model, making cycle conditions irrelevant for our work. When $\delta > 0$, spanning forests conditions must be resolved using the following method. The first step is to choose a spanning forest for the chemical network, and then choose an “orientation” for that spanning forest. By orientation, we mean a particular (but arbitrary) direction (forward or reverse) for each reversible reaction in the spanning forest. Once this is done, we write the following expression^48^:

24 $$\begin{aligned} \sum _{f} \alpha _{f}s_{f}&=  0, \end{aligned}$$

where the sum is over all the reactions *f* in the oriented forest, $\alpha _{f}$ are unknown coefficients, and $s_{f}$ are the stoichiometric vector corresponding to each reaction. The above gives a series of linear equations that can be solved for the coefficients $\alpha _{f}$. The system is underdetermined, meaning that we can choose some of the $\alpha _{f}$ coefficients arbitrarily. The point to note is that it is possible to choose $\delta$ linearly independent solutions to Eq. (24) by choosing the $\alpha _{f}$ coefficients carefully. For each of the $\delta$ linearly independent solutions to Eq. (24), we write^48^:

25 $$\begin{aligned} \prod _{f} k_{f}^{\alpha _{f}}&=  \prod _{f} r_{f}^{\alpha _{f}}, \end{aligned}$$

where the $k_{f}$’s and $r_{f}$’s are the forward and reverse reaction rates for the reactions in the spanning forest, respectively. Equation (25) gives the desired constraints on the rate constants, and it is valid for any chemical model.

In the following, we first apply the general formalism discussed above to the uncatalyzed fully connected Smoluchowski model, and then generalize to the catalyzed fully connected homogeneous chiral Smoluchowski model. To find the number of thermodynamic constraints that need to be satisfied, it is necessary to compute $\gamma$ and $\delta$. Since the model is fully connected, it is possible to obtain analytic expressions for $\gamma$ and $\delta$ in terms of *N*. First, we write the total number of reaction pairs corresponding to Eq. (1) as:

26 $$\begin{aligned} p&=  \sum _{n=1}^{N} p_{n}, \end{aligned}$$

where $p_{n}$ is the number of reaction pairs that gives the product C$_{n}$. For a fully connected chemistry, $p_{n}$ can be written as:

27 $$\begin{aligned} p_{n}&=  \sum _{i \le j, i+j=n} 1 \;=\; \left\{ \begin{array}{cl} \frac{n}{2} &{}\quad \text{(n } \text{ even) } \\ &{} \\ \frac{n-1}{2} &{}\quad \text{(n } \text{ odd) } \end{array} \right. \end{aligned}$$

Combining Eqs. (26) and (27), we obtain:

28 $$\begin{aligned} p&=  \left\{ \begin{array}{cl} \frac{N^{2}}{4} &{}\quad \text{(N } \text{ even) } \\ &{} \\ \frac{N^{2}-1}{4} &{}\quad \text{(N } \text{ odd) } \end{array} \right. \end{aligned}$$

The number of complexes is related to the number of pairs, but is not quite double the number of pairs because some reactions give the same product. For a specific *n*, there is one complex for the product $C_{n}$, and $p_{n}$ reaction pairs that gives this product, for a total of $1 + p_{n}$ complexes for this *n*. The total number of complexes is thus:

29 $$\begin{aligned} c&=  \sum _{n=2}^{N} \left( 1 + p_{n}\right) \;=\; \left\{ \begin{array}{ll} N - 1 + \frac{N^{2}}{4} &{}\quad \text{(N } \text{ even) }\;\; \\ &{} \\ N - 1 + \frac{N^{2}-1}{4} &{}\quad \text{(N } \text{ odd) }\;\; \end{array} \right. \end{aligned}$$

The number of linkage classes is directly proportional to the number of possible products: each product C$_{n}$ acts as a new “center” to which possible reactions connect to. The only exception is C$_{1}$, since it is a monomer and no reaction involving two molecules can give C$_{1}$. Thus C$_{1}$ cannot act as a “center”, and the number of possible linkage classes is:

30 $$\begin{aligned} l&=  N - 1. \end{aligned}$$

The rank of the stoichiometric matrix corresponds to the number of linearly independent reaction vectors in the stoichiometric matrix. In the Smoluchowski model, the set of reactions involving C$_{1}$ (i.e. $\textrm{C}_{1} + \textrm{C}_{1} \rightleftharpoons \textrm{C}_{2}$, $\textrm{C}_{1} + \textrm{C}_{2} \rightleftharpoons \textrm{C}_{3}$, etc) produce linearly independent reaction vectors in the stoichiometric matrix, and can also serve as a basis. Thus all other reaction vectors can be expressed as a linear combination of those basis vectors. This implies that the rank of the stoichiometric matrix is equal to the number of reactions involving C$_{1}$:

31 $$\begin{aligned} s &=  {} N - 1. \end{aligned}$$

Using Eqs. (28)–(31), we can compute $\gamma$ and $\delta$ for the uncatalyzed fully connected Smoluchowski model:

32 $$\begin{aligned} \gamma &=  0, \end{aligned}$$

33 $$\begin{aligned} \delta &=  \left\{ \begin{array}{cl} 1 - N + \frac{N^{2}}{4} &{}\quad \text{(N } \text{ even) } \\ &{} \\ 1 - N + \frac{N^{2}-1}{4} &{}\quad \text{(N } \text{ odd) } \end{array} \right. \end{aligned}$$

Including catalysis to the above results can be done by inspection. Assuming that *q* reactions in the fully connected Smoluchowski model are now catalyzed by a molecule present in the system, it can be shown that relevant quantities are modified in the following way: $p\rightarrow p + q$, $c\rightarrow c + 2q$, $l\rightarrow l+ q$, $s\rightarrow s$. Consequently, we have that $\gamma \rightarrow \gamma$ and $\delta \rightarrow \delta + q$. Thus having *q* catalyzed reactions in the system only adds *q* spanning forest conditions that need to be satisfied in order for the chemistry to be consistent with thermodynamics. But thanks to the parametrization (5)–(6) and the form of spanning forest constraints (see Eq. (25)), we can see that any additional constraints coming from catalysis are automatically satisfied. Thus Eqs. (32)–(33) still hold in the presence of catalysis.

Considering now the chiral fully connected Smoluchowski model in which the L and D sectors interact only through enantiomerization of one food molecule, the relevant quantities are modified in the following way: $p \rightarrow 2p + 1$, $c \rightarrow 2c + 2$, $l \rightarrow 2l + 1$, $s \rightarrow 2s + 1$, where the factors of two come from the doubling of reactions (L and D), and the added factors (either 1 or 2) come from the enantiomerization reaction. Consequently, we have that $\gamma \rightarrow 2\gamma$ and $\delta \rightarrow 2\delta$. We thus see that the number of constraints doubles when the model is made chiral, although this doubling is only apparent. Since the rate constants in each sector are the same (unless there is a chiral bias), the constraints in both sectors must be numerically the same. Thus making the model chiral does not produce additional constraints on top of those required by Eqs. (32)–(33).

Equations (32)–(33) imply that the rate constants of the fully connected Smoluchowski model must satisfy a certain number of constraints in order for it to be consistent with thermodynamics. In the simplified situation where all forward (reverse) reaction rates are equal, the spanning forest constraints (25) take the form:

34 $$\begin{aligned} k^{\sum _{f}\alpha _{f}} &=  r^{\sum _{f}\alpha _{f}}. \end{aligned}$$

The $\alpha _{f}$’s obey a sum rule in the Smoluchowski model:

35 $$\begin{aligned} \sum _{f} \alpha _{f} &=  0 \end{aligned}$$

[To obtain the above sum rule, one starts with Eq. (24) and multiply both sides by a column vector of size *N* entirely made of ones:

36 $$\begin{aligned} \sum _{f} \alpha _{f} \left( \sum _{s=1}^{N} S_{fs}\right) &=  0, \end{aligned}$$

where $S_{fs}$ is the stoichiometric matrix of the system. Since all reactions in the Smoluchowski model are $2\rightarrow 1$ aggregation reactions, we have $\sum _{s=1}^{N} S_{fs} = -1 \;\forall f$, leading directly to the sum rule (35).] Finally, substituting the sum rule (35) into Eq. (34), we obtain that all thermodynamic constraints in the fully connected Smoluchowski model with equal rate constants are automatically satisfied. Note that a similar result can be obtained using simpler arguments involving the Gibbs free energy (see the Appendix in Ref.^50^).

## Numerical results

We search for mirror symmetry breaking in the fully connected, homogeneous, and dimensionless chiral Smoluchowski model by numerically integrating Eqs. (16)–(17) using the NDSolve built-in command in Mathematica. To perform the numerical integration, it is necessary to specify a set of dimensionless parameters (*N*, $\tilde{k}$, $\tilde{r}$, $\tilde{f}$), a particular catalytic configuration (see below for examples), and initial concentrations for all molecules in the system. In the spirit of origin of life research, we assume that only the smallest building block (C$_{1}^\mathrm{L,D}$) is present initially, and larger molecules slowly build up over time (i.e. $C_{n}^\mathrm{L,D}(t=0) = 0 \;\forall n \ge 2$). We further assume that there is an imbalance in the initial concentrations of C$_{1}^\mathrm{L}$ and C$_{1}^\mathrm{R}$. This initial imbalance in the two handedness can be due to various physical or chemical mechanisms, such as the ever-present statistical fluctuations in otherwise symmetric chemical mixtures^16,51^, the presence of circularly polarized light in star-forming regions^52–54^, the weak nuclear force^55,56^, or delivery via meteorites^57,58^. The source and magnitude of the imbalance is not important for this paper, as long at it is nonzero (the magnitude influences the time at which homichirality sets in, but not its appearance). We use $\tilde{C}_{1}^\mathrm{L}(t=0) = 1.1$ and $\tilde{C}_{1}^\mathrm{D}(t=0) = 0.9$ in all numerical simulations in the following (corresponding to an initial imbalance of $10\%$). Note that we tested several catalytic configurations with much lower initial imbalances (one part in $10^{-14}$), and obtained similar results.

**Figure 1:**
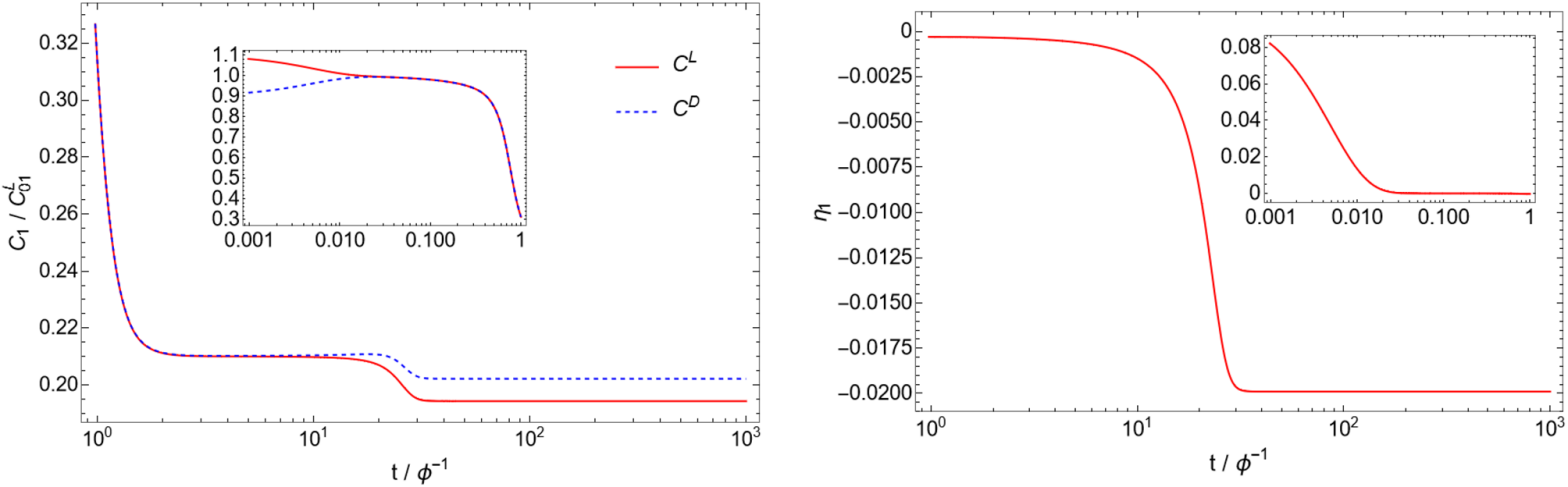
Time evolution of the C$_{1}$ concentration (left), and the enantiomeric excess $\eta _{1}$ (right), for the catalytic configuration D4 (see Table 2) with parameters $N=4$, $\tilde{k} = 0.1$, $\tilde{r} = 0.001$, and $\tilde{f} = 100$. Time is measured in units of residence time ($\phi ^{-1}$), and concentrations are measured in units of the initial monomer concentration ($C_{01}^\mathrm{L}$) (see Eqs. 14–15). Insets represent the same plot with a different time range.

**Figure 2:**
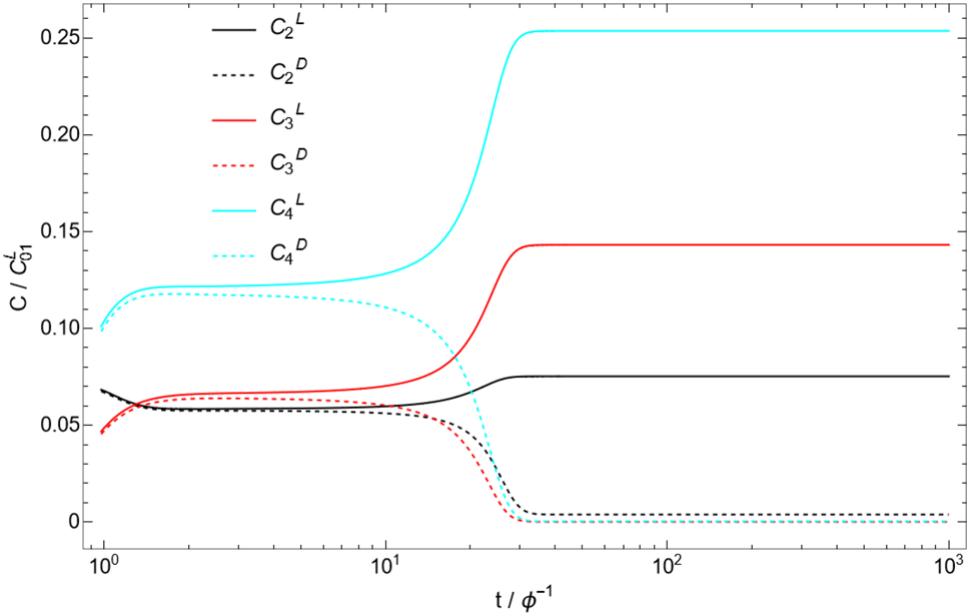
Time evolution of the C$_{2}$, C$_{3}$, and C$_{4}$ concentrations for the catalytic configuration D4 (see Table 2) with parameters $N=4$, $\tilde{k} = 0.1$, $\tilde{r} = 0.001$, and $\tilde{f} = 100$. Time is measured in units of residence time ($\phi ^{-1}$), and concentrations are measured in units of the initial monomer concentration ($C_{01}^\mathrm{L}$) (see Eqs. 14–15).

**Figure 3:**
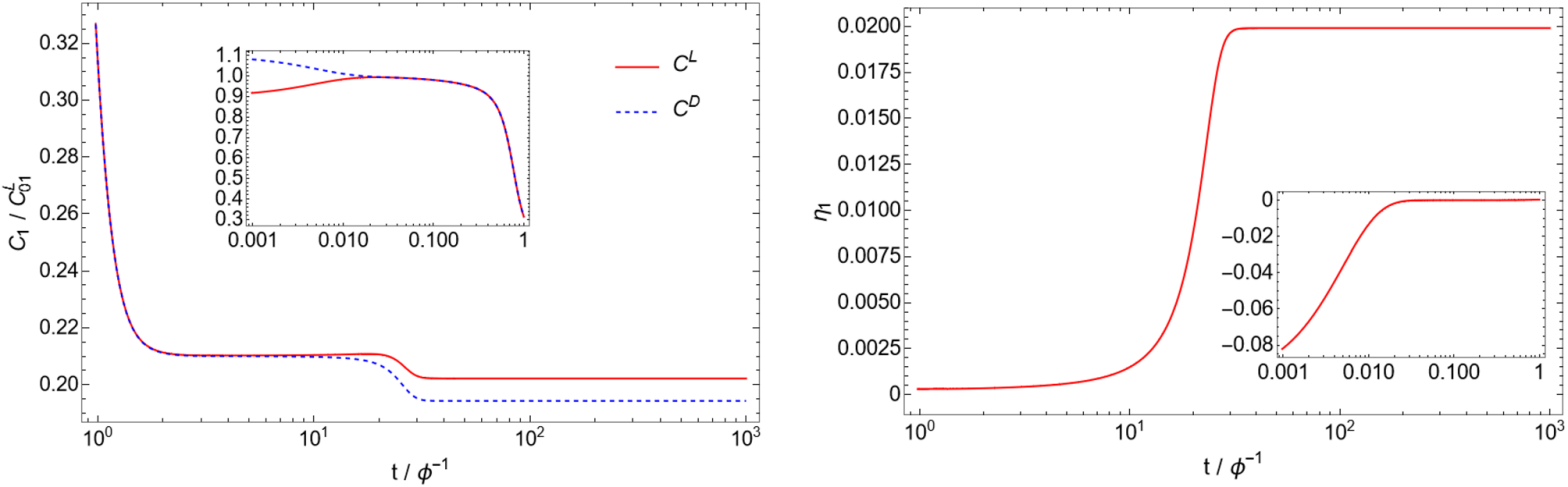
Time evolution of the C$_{1}$ concentration (left), and the enantiomeric excess $\eta _{1}$ (right) with parameters identical to the ones of Fig. 1, but with inverted initial conditions ($\tilde{C}_{1}^\mathrm{L}(t=0) = 0.9$ and $\tilde{C}_{1}^\mathrm{D}(t=0) = 1.1$). As expected, the behavior is the mirror image of the one in Fig. 1.

Figure 1 shows the time evolution of the C$_{1}$ molecule concentration and the corresponding enantiomeric excess:

37 $$\begin{aligned} \eta _{i}\equiv & {} \frac{\tilde{C}_{i}^\mathrm{L} - \tilde{C}_{i}^\mathrm{D}}{\tilde{C}_{i}^\mathrm{L} + \tilde{C}_{i}^\mathrm{D}} \end{aligned}$$

for a particular catalytic configuration called an autocatalytic set (catalytic configuration D4 with $N=4$, described below). Note that the enantiomeric excess is not well-defined when both concentrations are zero, and is thus not meaningful at $t=0$ in our simulations (for $n\ge 2$). Due to the asymmetric initial condition, the enantiomeric excess $\eta _{1}$ is nonzero at $t=0$, but this excess is almost completely erased in a time $\tilde{t} \approx 1/\tilde{f} = 0.01$ due to enantiomerization. At this stage, it is the stability of the racemic state that determines if the system will break mirror symmetry or not. In the case of the autocatalytic set simulated here, the nonlinearity of the system is sufficient to make the racemic state unstable. Due to this instability, the enantiomeric excess increases dramatically between $\tilde{t}\approx 10$ and $\tilde{t}\approx 30$, until it reaches a stable value of $\eta _{1} \approx -0.020$. The other molecules in the system (C$_{2}$, C$_{3}$, C$_{4}$) behave in a similar way (see Fig. 2), with different final enantiomeric excesses ($\eta _{2} = 0.903$, $\eta _{3} = 0.999$, $\eta _{4} = 0.998$). The time evolution shown in Fig. 1 is typical of simulations where mirror symmetry is broken. Simulations in which mirror symmetry is not broken only exhibit the racemization phase where the initial enantiomeric excess goes to zero in a time $\tilde{t} \approx 1/\tilde{f}$. As a check of the numerics, we also ran the simulation with inverted initial conditions ($\tilde{C}_{1}^\mathrm{L}(t=0) = 0.9$ and $\tilde{C}_{1}^\mathrm{D}(t=0) = 1.1$), and obtained the expected inverted behavior for the concentrations and enantiomeric excess (see Fig. 3).

**Figure 4:**
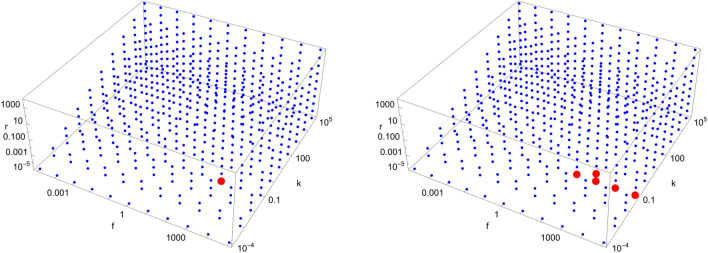
Scan of parameter space ($\tilde{k}$, $\tilde{r}$, $\tilde{f}$) for the D3 catalytic configuration with $N=3$ (left) and $N=40$ (right). Red (blue) dots indicate parameter values for which the enantiomeric excess is greater (lesser) than 0.5. The parameter range ($\tilde{k}$, $\tilde{r}$, $\tilde{f}$) for the red region is $\left( 10^{-1}, 10^{-5}, 10^{3}\right)$ (left) and $\left( 10^{-1}, \left[ 10^{-5},10^{-4}\right] , \left[ 10^{2},10^{5}\right] \right)$ (right), respectively.

**Figure 5:**
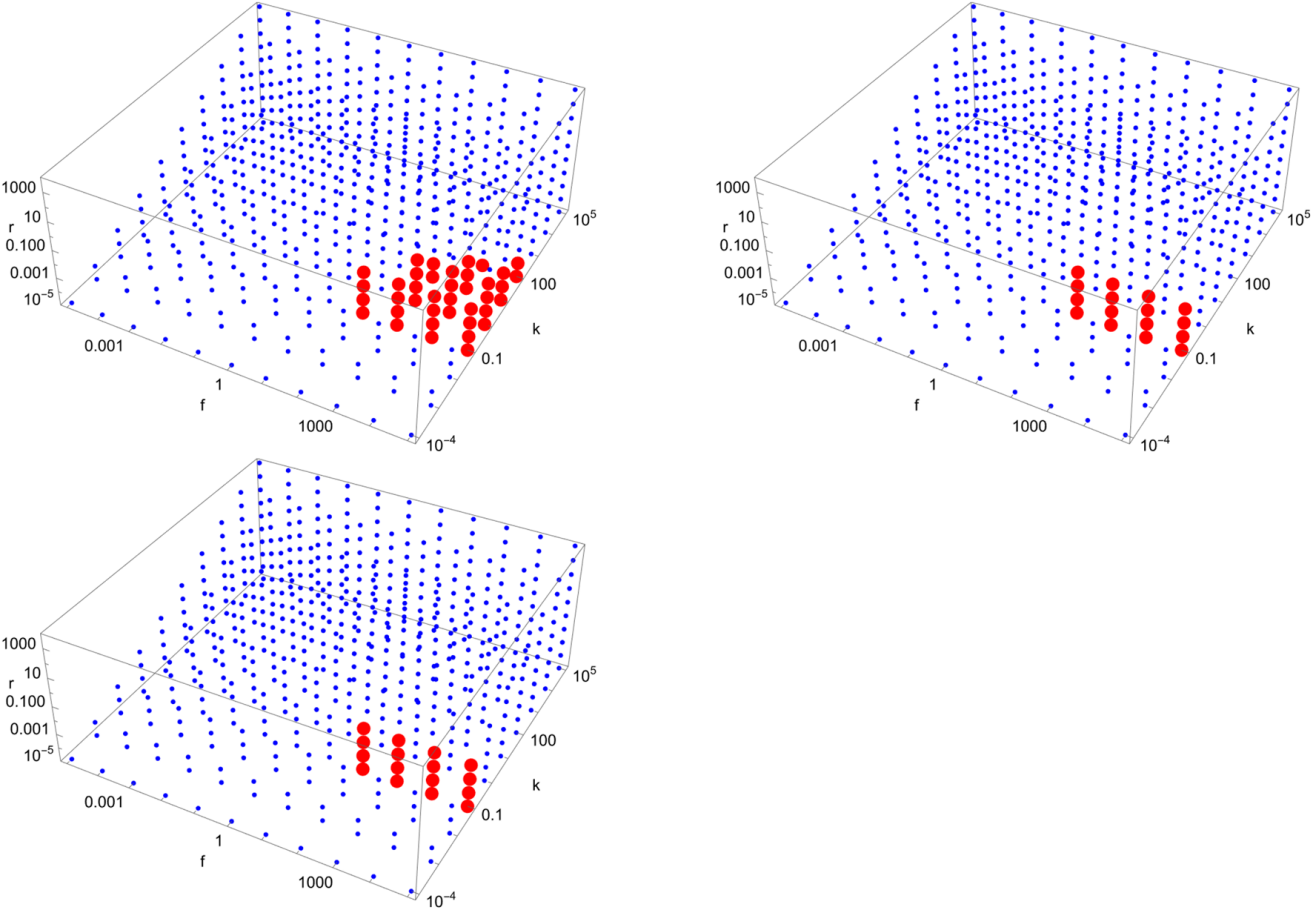
Scan of parameter space ($\tilde{k}$, $\tilde{r}$, $\tilde{f}$) for the D4 catalytic configuration with $N=4$ (top left), $N=10$ (top right), and $N=40$ (bottom left). Red (blue) dots indicate parameter values for which the enantiomeric excess is greater (lesser) than 0.5. The parameter range ($\tilde{k}$, $\tilde{r}$, $\tilde{f}$) for the red region is $\left( \left[ 10^{-1},10^{2}\right] , \left[ 10^{-5},10^{-2}\right] , \left[ 10^{2},10^{5}\right] \right)$ (top left), $\left( 10^{-1}, \left[ 10^{-5},10^{-2}\right] , \left[ 10^{2},10^{5}\right] \right)$ (top right) and $\left( 10^{-1}, \left[ 10^{-5},10^{-2}\right] , \left[ 10^{2},10^{5}\right] \right)$ (bottom left), respectively.

**Figure 6:**
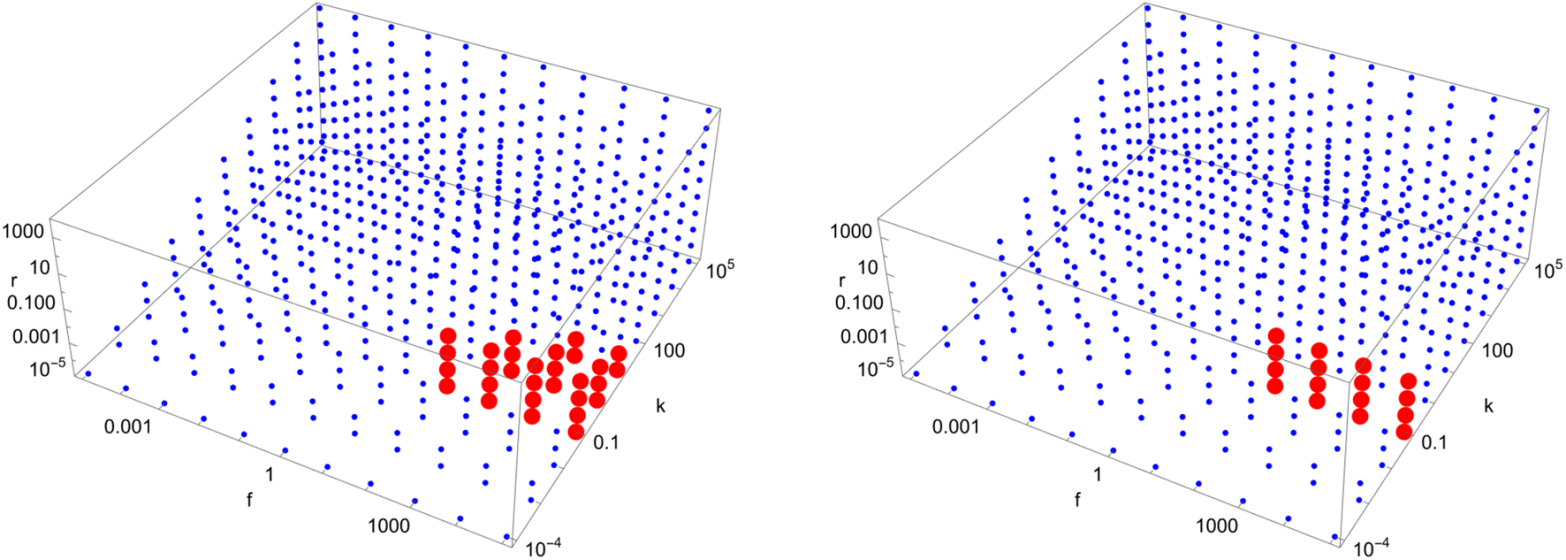
Scan of parameter space ($\tilde{k}$, $\tilde{r}$, $\tilde{f}$) for the D5 catalytic configuration with $N=4$ (left) and $N=40$ (right). Red (blue) dots indicate parameter values for which the enantiomeric excess is greater (lesser) than 0.5. The parameter range ($\tilde{k}$, $\tilde{r}$, $\tilde{f}$) for the red region is $\left( \left[ 10^{-1},10^{1}\right] , \left[ 10^{-5},10^{-2}\right] , \left[ 10^{2},10^{5}\right] \right)$ (left) and $\left( 10^{-1}, \left[ 10^{-5},10^{-2}\right] , \left[ 10^{2},10^{5}\right] \right)$ (right), respectively.

**Figure 7:**
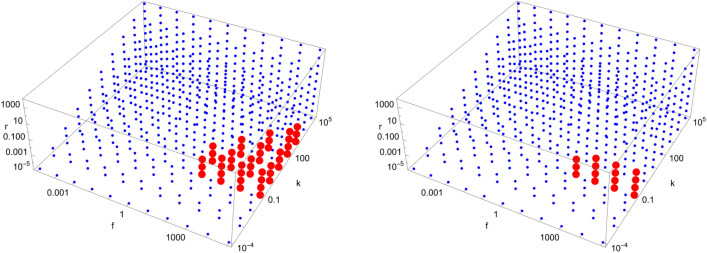
Scan of parameter space ($\tilde{k}$, $\tilde{r}$, $\tilde{f}$) for the D6 catalytic configuration with $N=4$ (left) and $N=40$ (right). Red (blue) dots indicate parameter values for which the enantiomeric excess is greater (lesser) than 0.5. The parameter range ($\tilde{k}$, $\tilde{r}$, $\tilde{f}$) for the red region is $\left( \left[ 10^{-1},10^{3}\right] , \left[ 10^{-5},10^{-2}\right] , \left[ 10^{2},10^{5}\right] \right)$ (left) and $\left( 10^{-1}, \left[ 10^{-5},10^{-2}\right] , \left[ 10^{2},10^{5}\right] \right)$ (right), respectively.

**Figure 8:**
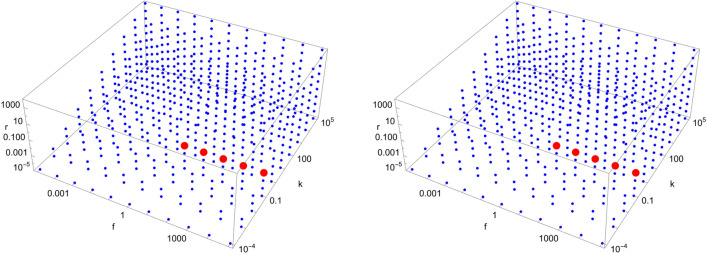
Scan of parameter space ($\tilde{k}$, $\tilde{r}$, $\tilde{f}$) for the D7 catalytic configuration with $N=4$ (left) and $N=40$ (right). Red (blue) dots indicate parameter values for which the enantiomeric excess is greater (lesser) than 0.5. The parameter range ($\tilde{k}$, $\tilde{r}$, $\tilde{f}$) for the red region is $\left( 10^{-1}, 10^{-2}, \left[ 10^{1},10^{5}\right] \right)$ (left) and $\left( 10^{-1}, 10^{-2}, \left[ 10^{1},10^{5}\right] \right)$ (right), respectively.

**Figure 9:**
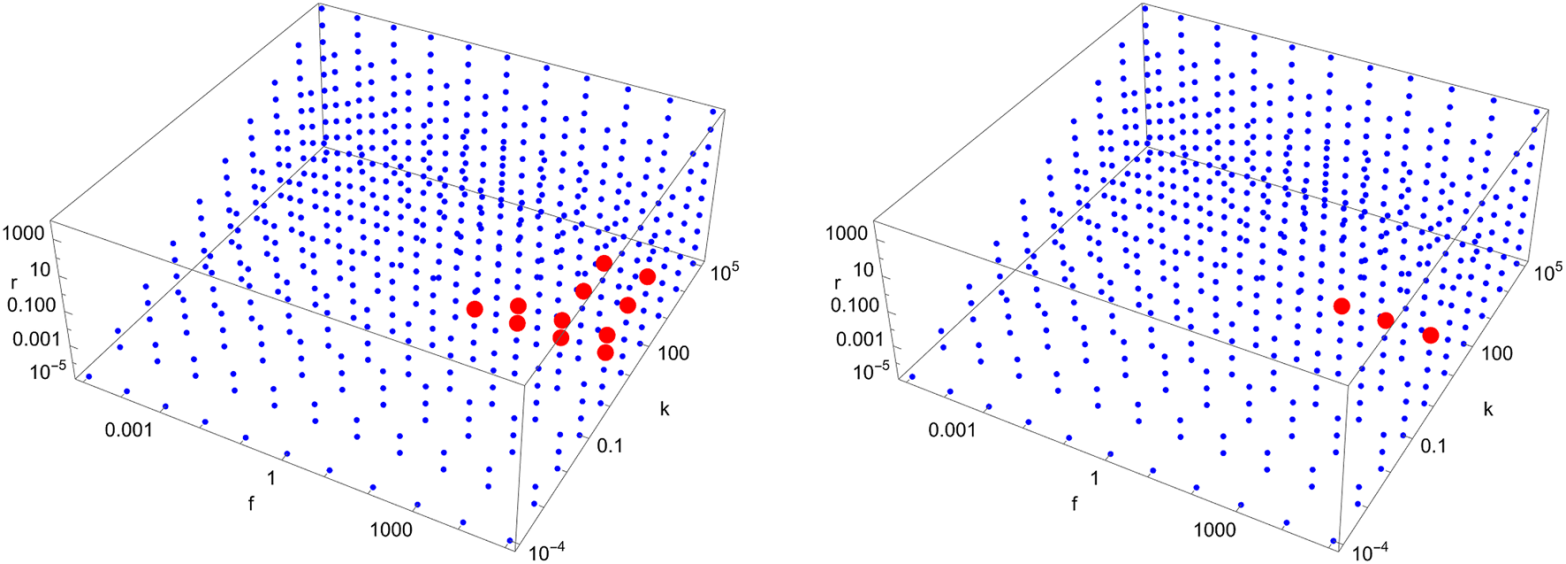
Scan of parameter space ($\tilde{k}$, $\tilde{r}$, $\tilde{f}$) for the D8 catalytic configuration with $N=4$ (left) and $N=40$ (right). Red (blue) dots indicate parameter values for which the enantiomeric excess is greater (lesser) than 0.5. The parameter range ($\tilde{k}$, $\tilde{r}$, $\tilde{f}$) for the red region is $\left( \left[ 10^{0},10^{2}\right] , \left[ 10^{-2},10^{-1}\right] , \left[ 10^{2},10^{5}\right] \right)$ (left) and $\left( 10^{0}, 10^{-1}, \left[ 10^{3},10^{5}\right] \right)$ (right), respectively.

**Figure 10:**
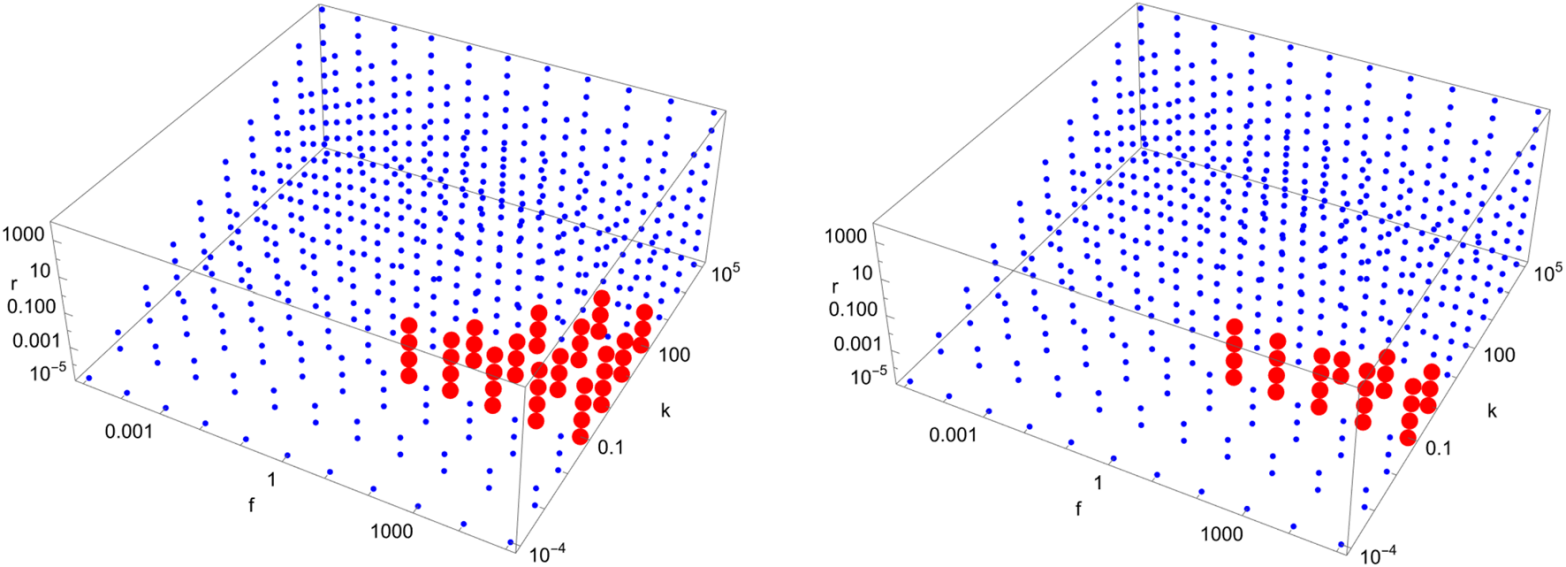
Scan of parameter space ($\tilde{k}$, $\tilde{r}$, $\tilde{f}$) for the D9 catalytic configuration with $N=4$ (left) and $N=40$ (right). Red (blue) dots indicate parameter values for which the enantiomeric excess is greater (lesser) than 0.5. The parameter range ($\tilde{k}$, $\tilde{r}$, $\tilde{f}$) for the red region is $\left( \left[ 10^{-1},10^{2}\right] , \left[ 10^{-5},10^{-2}\right] , \left[ 10^{1},10^{5}\right] \right)$ (left) and $\left( \left[ 10^{-1},10^{0}\right] , \left[ 10^{-5},10^{-2}\right] , \left[ 10^{1},10^{5}\right] \right)$ (right), respectively.

**Figure 11:**
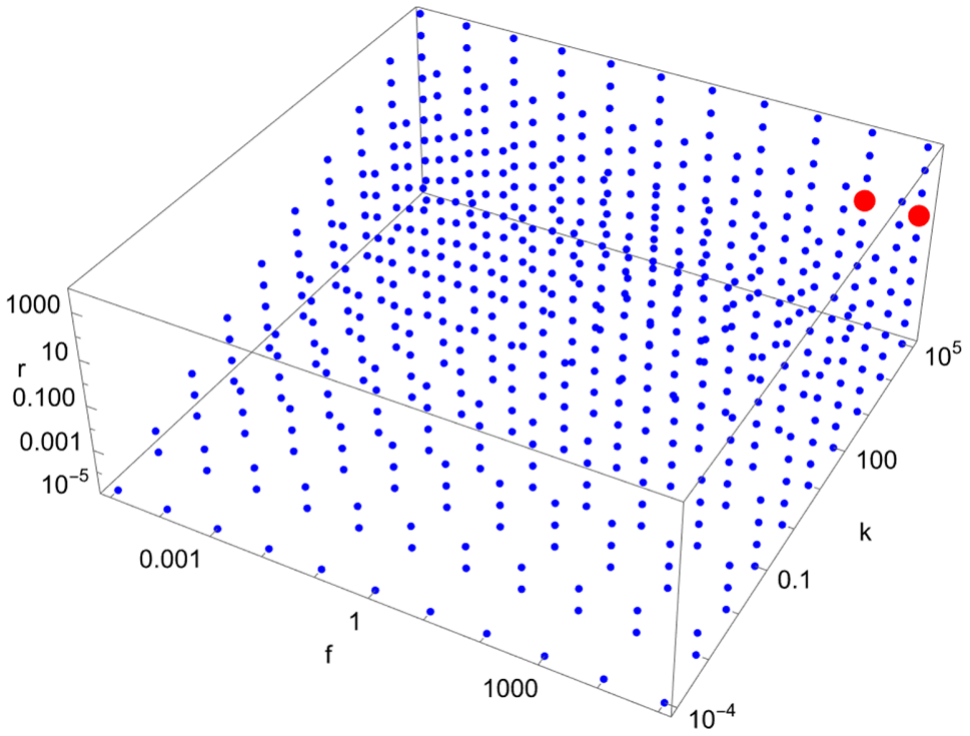
Scan of parameter space ($\tilde{k}$, $\tilde{r}$, $\tilde{f}$) for the D11 catalytic configuration with $N=40$. Red (blue) dots indicate parameter values for which the enantiomeric excess is greater (lesser) than 0.5. The parameter range ($\tilde{k}$, $\tilde{r}$, $\tilde{f}$) for the red region is $\left( 10^{5}, 10^{1}, \left[ 10^{4},10^{5}\right] \right)$.

**Figure 12:**
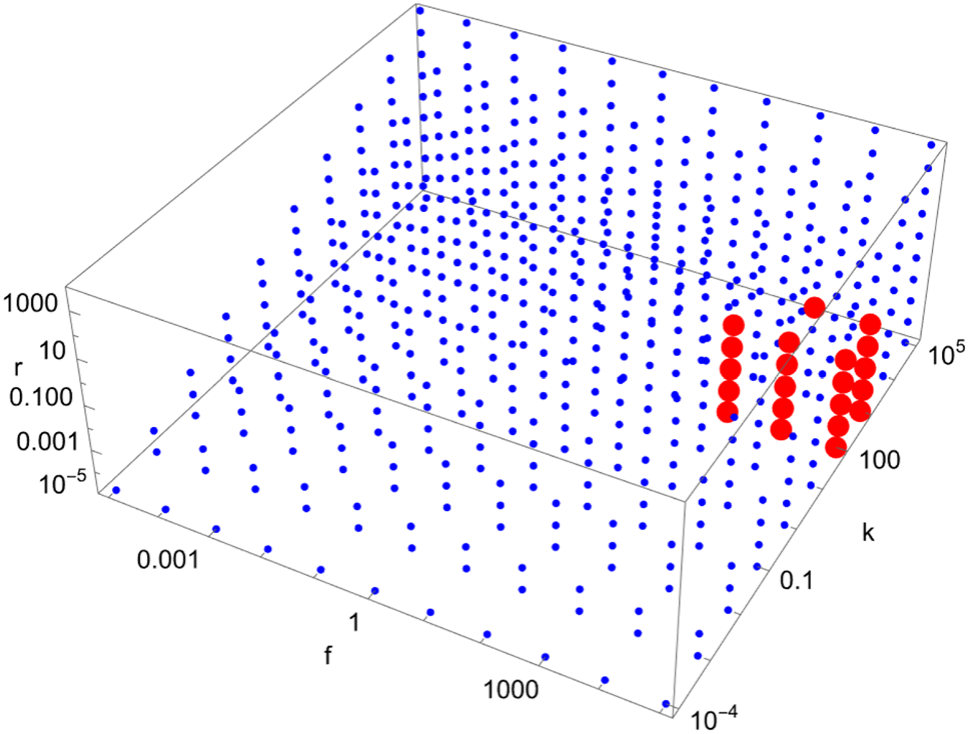
Scan of parameter space ($\tilde{k}$, $\tilde{r}$, $\tilde{f}$) for the D12 catalytic configuration with $N=40$. Red (blue) dots indicate parameter values for which the enantiomeric excess is greater (lesser) than 0.5. The parameter range ($\tilde{k}$, $\tilde{r}$, $\tilde{f}$) for the red region is $\left( \left[ 10^{2},10^{3}\right] , \left[ 10^{-5},10^{-1}\right] , \left[ 10^{3},10^{5}\right] \right)$.

**Figure 13:**
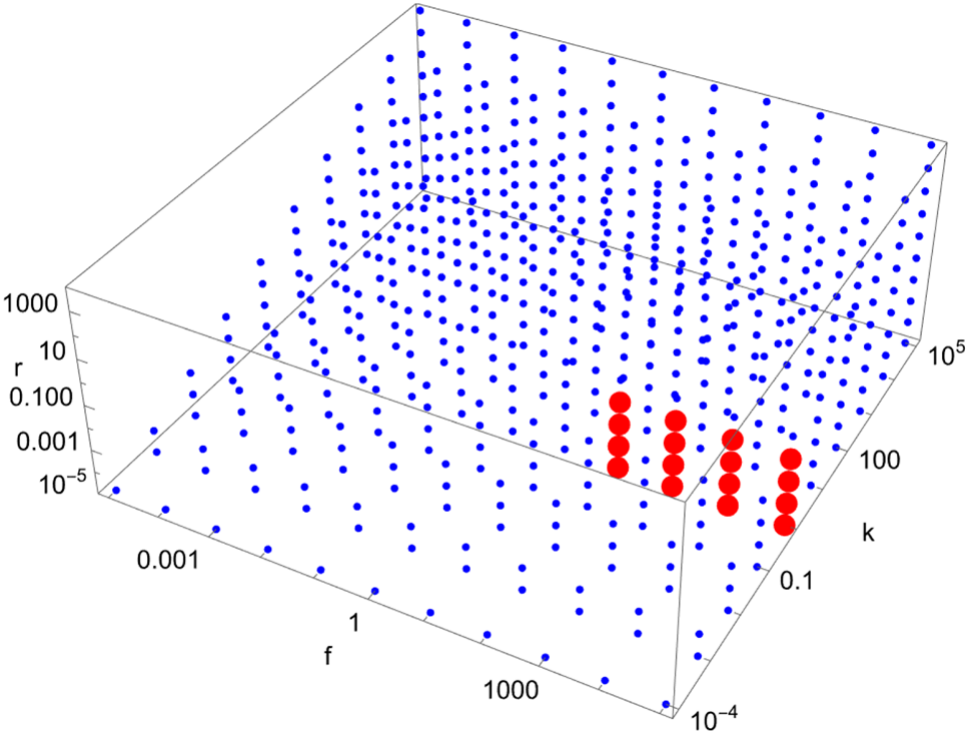
Scan of parameter space ($\tilde{k}$, $\tilde{r}$, $\tilde{f}$) for the D35 catalytic configuration with $N=40$. Red (blue) dots indicate parameter values for which the enantiomeric excess is greater (lesser) than 0.5. The parameter range ($\tilde{k}$, $\tilde{r}$, $\tilde{f}$) for the red region is $\left( 10^{0}, \left[ 10^{-5},10^{-2}\right] , \left[ 10^{2},10^{5}\right] \right)$.

Figures 4, 5, 6, 7, 8, 9, 10, 11, 12 and 13 show scans of parameter space ($\tilde{k}$, $\tilde{r}$, $\tilde{f}$) for different catalytic configurations and number of molecules. Since rate constants in chemistry take values spanning many orders of magnitude, we logarithmically choose our rate constants to be $\tilde{k} = 10^{i}$, $\tilde{r} = 10^{j}$, $\tilde{f} = 10^{k}$, where *i*, *j*, *k* are integers between $-5$ and 5, with the constraint that $\tilde{k} > \tilde{r}$ (i.e. forward rate constants are larger than reverse rate constants). We also take all catalytic strengths to be the same ($\kappa _{s}^{ij} = 1000\; \forall i,j,s$). For each value of the parameters, we numerically integrate Eqs. (16)–(17) for $10^{12}$ time units to allow the system sufficient time to reach stationary concentrations. The enantiomeric excess of the largest molecule in the system is then read at $t=10^{12}$. If the excess is greater (less) than a certain threshold (taken to be 0.5 here), a red (blue) dot is put in the figure. Each figure contains a total of 605 different simulations for the same catalytic configuration and number of molecules. It takes about $12-16$ hours to produce each figure on a standard laptop when $N=40$.

Tables 2 and 3 show a summary of our simulation results for various catalytic configurations (labeled D0 to D59) and total number of molecules. Catalyzed reactions in each catalytic configurations are indicated in the table, with the notation “i-j-k” corresponding to the reaction C$_{i}+$C$_{j}+$C$_{k} \rightleftharpoons$ C$_{i+j}+$C$_{k}$. For each catalytic configuration and *N*, we indicate if spontaneous mirror symmetry breaking (SMSB) has been achieved or not (i.e. $\eta _{N} \ge 0.5$). Note that some catalytic configurations break mirror symmetry for a larger fraction of parameter space, which can be seen by counting the number of red dots in Figs. 4, 5, 6, 7, 8, 9, 10, 11, 12 and 13 (keeping in mind that the scale is logarithmic). We report the extent to which mirror symmetry is broken in the last column of Tables 2 and 3.

**Table 2:** Summary of results for different catalytic configurations (part 1).

Cat. config.	*N*	Catalyzed reactions	Comments	SMSB?
D0	4	None		No
D0	50	None		No
D0	100	None		No
D1	2	1-1-2	One replicator	No
D1	4	1-1-2	One replicator	No
D1	40	1-1-2	One replicator	No
D2	3	1-1-3, 1-2-1	Autocatalytic set with self-catalyzed reactions	No
D2	4	1-1-3, 1-2-1	Autocatalytic set with self-catalyzed reactions	No
D2	40	1-1-3, 1-2-1	Autocatalytic set with self-catalyzed reactions	No
D3	3	1-1-2, 1-2-3	Two replicators, autocatalytic set (not hypercycle)	Yes (1 dot)
D3	4	1-1-2, 1-2-3	Two replicators, autocatalytic set (not hypercycle)	No
D3	40	1-1-2, 1-2-3	Two replicators, autocatalytic set (not hypercycle)	Yes (5 dots)
D4	4	1-1-3, 1-2-4, 1-3-2, 2-2-1	Autocatalytic set	Yes (39 dots)
D4	10	1-1-3, 1-2-4, 1-3-2, 2-2-1	Autocatalytic set	Yes (16 dots)
D4	40	1-1-3, 1-2-4, 1-3-2, 2-2-1	Autocatalytic set	Yes (16 dots)
D5	4	1-1-3, 1-2-4, 1-3-2	Same as D4, minus 2-2-1	Yes (29 dots)
D5	40	1-1-3, 1-2-4, 1-3-2	Same as D4, minus 2-2-1	Yes (16 dots)
D6	4	1-1-3, 1-2-4, 2-2-1	Same as D4, minus 1-3-2	Yes (46 dots)
D6	40	1-1-3, 1-2-4, 2-2-1	Same as D4, minus 1-3-2	Yes (15 dots)
D7	4	1-1-3, 1-3-2, 2-2-1	Same as D4, minus 1-2-4	Yes (5 dots)
D7	40	1-1-3, 1-3-2, 2-2-1	Same as D4, minus 1-2-4	Yes (5 dots)
D8	4	1-2-4, 1-3-2, 2-2-1	Same as D4, minus 1-1-3	Yes (11 dots)
D8	40	1-2-4, 1-3-2, 2-2-1	Same as D4, minus 1-1-3	Yes (3 dots)
D9	4	1-1-3, 1-2-4	Same as D4, minus 1-3-2 and 2-2-1	Yes (48 dots)
D9	40	1-1-3, 1-2-4	Same as D4, minus 1-3-2 and 2-2-1	Yes (28 dots)
D9a	4	1-1-3	Same as D4, minus 1-2-4, 1-3-2 and 2-2-1	No
D9a	40	1-1-3	Same as D4, minus 1-2-4, 1-3-2 and 2-2-1	No
D9b	4	1-2-4	Same as D4, minus 1-1-3, 1-3-2 and 2-2-1	No
D9b	40	1-2-4	Same as D4, minus 1-1-3, 1-3-2 and 2-2-1	No
D10	40	10-10-30, 10-20-40,	Autocatalytic set (similar to D4)	No
		10-30-20, 20-20-10		
D11	40	1-36-40, 1-37-39,		Yes (2 dots)
		1-38-37, 1-39-38		
D12	40	1-1-40	Catalysis of early reaction by large molecules	Yes (21 dots)
D13	40	1-2-40	Catalysis of early reactions by large molecules	No
D14	40	1-3-40	Catalysis of early reactions by large molecules	No
D15	40	1-4-40	Catalysis of early reactions by large molecules	No
D16	40	2-2-40	Catalysis of early reactions by large molecules	No
D17	40	2-3-40	Catalysis of early reactions by large molecules	No
D18	40	2-4-40	Catalysis of early reactions by large molecules	No
D19	40	20-20-1	Catalysis of late reactions by small molecules	No
D20	40	15-25-1	Catalysis of late reactions by small molecules	No
D21	40	10-30-1	Catalysis of late reactions by small molecules	No
D22	40	5-35-1	Catalysis of late reactions by small molecules	No

**Table 3:** Summary of results for different catalytic configurations (part 2).

Cat. config.	*N*	Catalyzed reactions	Comments	SMSB?
D23-D27	40	Random	Randomly chosen catalyzed reactions (5,10,$\dots$,30 reactions)	No
D28-D32	40	Random	Random autocatalytic set adapted from D23-D27	No
D33	40	Random	Same as D23 with one reaction replaced by C$_{1}$+C$_{1}$	No
D34	40	Random	Same as D24 with one reaction replaced by C$_{1}$+C$_{1}$	No
D35	40	Random	Same as D25 with one reaction replaced by C$_{1}$+C$_{1}$	Yes (16 dots)
D36	40	Random	Same as D26 with one reaction replaced by C$_{1}$+C$_{1}$	No
D37	40	Random	Same as D27 with one reaction replaced by C$_{1}$+C$_{1}$	No
D38-D42	40	Random	Same as D28-D32 with one reaction replaced by C$_{1}$+C$_{1}$	No
D43-D47	40	Random	Same as D23-D27 with one reaction replaced by C$_{1}$+C$_{2}$	No
D48-D52	40	Random	Same as D28-D32 with one reaction replaced by C$_{1}$+C$_{2}$	No
D53	5	1-2-5		No
D53	40	1-2-5		No
D54	6	1-2-6		No
D54	40	1-2-6		No
D55	10	1-2-10		No
D55	40	1-2-10		No
D56	20	1-2-20		No
D56	40	1-2-20		No
D57	4	1-1-2, 1-1-4, 2-2-4, 2-2-2	Hypercycle (with two replicators)	No
D57	40	1-1-2, 1-1-4, 2-2-4, 2-2-2	Hypercycle (with two replicators)	No
D58	6	1-1-2, 1-1-4, 2-2-4, 2-2-6,	Hypercycle (with three replicators)	No
		3-3-2, 3-3-6		
D58	40	1-1-2, 1-1-4, 2-2-4, 2-2-6,	Hypercycle (with three replicators)	No
		3-3-2, 3-3-6		
D59	16	2-2-4, 2-2-6, 3-3-6, 3-3-10,	Hypercycle (with four replicators)	No
		5-5-10, 5-5-16, 8-8-4, 8-8-16		
D59	40	2-2-4, 2-2-6, 3-3-6, 3-3-10,	Hypercycle (with four replicators)	No
		5-5-10, 5-5-16, 8-8-4, 8-8-16		

Some of the catalytic configurations studied in the present paper are motivated by origins-of-life and homochirality models in the literature. For instance, replicators (catalytic configurations D1, D3, D57-D59) refer to molecules that can replicate themselves, as in the prototypical Selkov-Gray-Scott reaction $U + 2V \rightarrow 3V$^59,60^, or the RNA molecule (e.g.^61^). Autocatalytic sets are networks of reactions in which all reactions in the network are catalyzed by a molecule present in the network^18,20^. Catalytic configuration D4 with $N=4$ is a good example of autocatalytic set, where all four possible reactions are catalyzed by one of the four molecules in the network. This is to be contrasted with other catalytic configurations (D10, D28-D32, D43-D47) in which only a subset of reactions form an autocatalytic set. The hypercycle (catalytic configurations D57-D59) is a particular type of autocatalytic set in which a set of reactions form a cycle, all molecules are replicators, and the product of each reaction catalyzes the next reaction in the cycle^25^. We also study examples (catalytic configurations D12-D18) where larger molecules catalyze the production of smaller molecules (as in the homochirality-generating polymerization toy model of Ref.^62^), or vice-versa (catalytic configurations D19-D22).

## Discussion

It is important to stress that the chiral Smoluchowski model studied here is simple, and only represents a tiny fraction of the complexity of chemical reaction networks. We justify the simplifications made (i.e fully connected, homogeneous) by the fact that they allow to study the parameter space of the model in a systematic way (i.e. scanning only three parameters $\tilde{k}$, $\tilde{r}$, $\tilde{f}$), and also allow to prove analytically that the model is consistent with thermodynamics. But this is far from being a full scan of parameter space. As a comparison, a non-fully connected, non-homogeneous model with $N=40$ would contain up to 400 reactions (see Eq. 28) that could be connected in an extremely large number of ways, each with its own forward and reverse reaction rate. Taking into account catalysis also adds complexity to the analysis. This is why we narrow down the possible catalytic properties of the network using inspiration from origin-of-life models, but again we make the simplification that all catalytic enhancement factors are equal. Thus the conclusions drawn here should not be taken as strict statements about chemical networks, but as suggestive chemical behaviors of potential interest to the homochirality and prebiotic chemistry communities.

The appearance of homochirality in a chemical system is generally attributed to the amplification of an initial chiral imbalance through (sufficiently) nonlinear interactions^15,16^. Unsurprisingly and in agreement with the literature, we observe no spontaneous mirror symmetry breaking in our model when no catalysis is present (see catalytic configuration D0 in Table 2). Typically, models found in the literature require additional ingredients to produce homochirality, such as quadratic or even higher order autocatalysis^1^ and mutual inhibition^63^. Our results show that it is possible to break mirror symmetry with *only* catalyzed reactions, showing that other ingredients are not necessary. This is important from the prebiotic chemistry point of view, since pure single-step autocatalysis is rare in chemistry^64^, and mutual inhibition (e.g. two molecules of opposite handedness reacting together to form an achiral product, epimerization, or any other heterochiral interaction) as found in Frank-like models is detrimental to molecules of life as we know it. For example, mutual inhibition would imply the formation of proteins with a mixture of L and D amino acids, which would not fold properly^65^.

Catalytic configuration D4 with $N=4$ is an autocatalytic set that strongly breaks mirror symmetry ($\eta _{4}\approx 0.99$) for a sizable part of parameter space (39 dots). It does so with only catalyzed reactions arranged in such a way that all reactions in the network are catalyzed by a molecule present in the network. This is a strong requirement for a chemical network, thus in the spirit of looking for minimal conditions for homochirality, we also investigated similar catalytic configurations with fewer catalyzed reactions than D4 (see catalytic configurations D5-D9b). Our results show mirror symmetry can still take place with only catalyzed reactions not arranged in an autocatalytic set. We also explored other autocatalytic set configurations with various number of catalyzed reactions (catalytic configurations D10 and D28-D32), and none of them break mirror symmetry. We thus conclude that autocatalytic sets are not necessary nor sufficient to break mirror symmetry.

Catalytic configuration D9 shows that only two catalyzed reactions are sufficient to strongly break mirror symmetry, even with $N=40$ (corresponding to 400 reactions in the chemical network). This is very minimal, and hints at the possibility that reactions C$_{1}$+C$_{1}$ $\rightleftharpoons$ C$_{2}$ and C$_{1}$+C$_{2}$ $\rightleftharpoons$ C$_{3}$ have special properties that are favorable to the breaking of mirror symmetry. To test this, we generated catalytic configurations with a variable number of randomly chosen catalyzed reactions (see catalytic configurations D23-D27), being careful not to include the reactions present in D9. None of those random catalytic configurations are able to break mirror symmetry. Slightly modified versions of D23-D27 obtained by replacing one reaction with one of the reactions in D9 (see catalytic configurations D33–D37, D43–D47) show one case of mirror symmetry breaking (see D35). We also generated various catalytic configurations in which a large molecule catalyzes an “early” reaction involving small molecules (see catalytic configurations D12-D18). Among those catalytic configurations, only the one containing the C$_{1}$+C$_{1}$ reaction breaks mirror symmetry. These results suggest that catalyzing the reaction C$_{1}$+C$_{1}$ $\rightleftharpoons$ C$_{2}$ helps in breaking mirror symmetry, although catalytic configuration D9a shows that it cannot be a definitive requirement. We are uncertain as to the reason why this particular reaction is favorable to the breaking of mirror symmetry, but it may be due to one of its distinguishing features: it involves the smallest molecule in the network (from which all other molecules are built), as well as the only molecule that is fed into the system and racemizes.

Our results on catalytic configurations involving simple replicators (catalytic configurations D1, D3) and replicators arranged in a hypercycle (catalytic configurations D57-D59) show that they are not particularly successful in breaking mirror symmetry (only D3 is able to, in a very small part of parameter space). This is contrary to the results of Refs.^66,67^, where it is shown that hypercycles strongly break mirror symmetry. A possible reason to explain this discrepancy is that the model considered in Refs.^66,67^ allows the production of enantiomers from achiral sources, which is not the case in our model. Another important difference is that the model presented in Refs.^66,67^ only includes reactions directly involved in the hypercycle, and nothing else. In our model, other non-catalyzed “background” reactions are also present.

We think this last point is relevant for origin-of-life research, since prebiotic chemistry was most likely “messy” and included many background reactions. Background reactions change the structure of the concentration equations (12)–(13), and may affect the stability of the racemic state. This can be clearly seen in catalytic configuration D3, where mirror symmetry is broken for $N=3$, is restored for $N=4$, and is broken again for $N=40$. In addition, the fraction of parameter space where mirror symmetry is broken decreases when *N* increases (see catalytic configurations D4-D9). Thus we conclude that one must exercise caution when embedding a particular model into a larger chemical network.

We note from Figs. 4, 5, 6, 7, 8, 9, 10, 11, 12 and 13 that mirror symmetry is broken in the same region of parameter space for almost all catalytic configurations. For most cases, we have $\tilde{f} \ge \tilde{k}$, corresponding to $f \ge C_{01}^{L} k$. This numerical observation implies that enantiomerization should be more efficient than the combination of feeding and forward rates for mirror symmetry breaking to occur. We also note that the limits $\phi \rightarrow 0$ and $\phi \rightarrow \infty$ (corresponding to $\tilde{k},\tilde{r},\tilde{f}\rightarrow \infty$ and $\tilde{k},\tilde{r},\tilde{f}\rightarrow 0$, respectively) do not break mirror symmetry, as expected.

**Figure 14:**
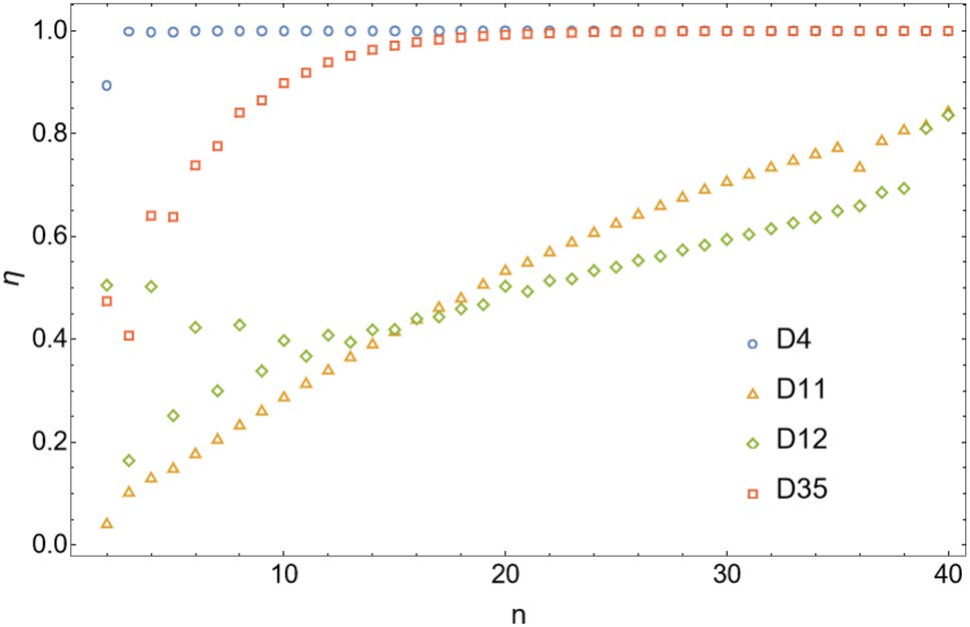
Enantiomeric excess as a function of the molecule size ($2 \le n \le N$) for the catalytic configurations D4, D11, D12, and D35 (see Table 2) with $N=40$.

Note also that the number of red dots in Figs. 4, 5, 6, 7, 8, 9, 10, 11, 12 and 13 depends in principle on the enantiomeric excess threshold chosen, but our experience with the model shows that the results are not very sensitive to it: either mirror symmetry for the largest molecule is largely broken (i.e. $\eta _{N} \gg 0.5$), or not at all (i.e. $\eta _{N}\approx 0$ within the precision of the numerical simulations). This conclusion does not necessarily hold for smaller molecules (with $n<N$), as the amount of enantiomeric excess typically increases with molecule size; see Fig. 14 for examples. This increase in enantiomeric excess with molecule size is also observed in Ref.^50^. The variation with *N* is nontrivial in some cases (as for catalytic configuration D12 in Fig. 14), but the general trend may be explained by the following argument. Writing $C^\mathrm{D} \equiv C$ and $C^\mathrm{L} \equiv C + \Delta C$, then $\eta = \Delta C/(2C + \Delta C) \approx \Delta C/(2C)$ when the excess is small compared to the “average” concentration of chemicals in the system. In a chemical network that builds larger molecules from smaller ones (as is the case here), the concentrations of larger molecules is typically much smaller than those of smaller molecules^34^. If the nonlinearities in the system produce a $\Delta C$ that is similar for different molecule sizes, then $\eta$ should increase with molecule size.

## Conclusion

In this paper we studied the generation of homochirality in a chiral version of the Smoluchowski aggregation-fragmentation model. We showed that it is possible to break mirror symmetry in various catalytic configurations that only involve a small number of catalyzed reactions and nothing else. In particular, our model does not require single-step autocatalysis or mutual inhibition to spontaneously break mirror symmetry. This may be of relevance for prebiotic chemistry, as single-step autocatalysis is rare, and mutual inhibition is not favorable to the appearance of the molecules of life as we know it (enzymes, RNA, DNA). Note that even if the underlying reasons as to why a specific catalytic configuration breaks mirror symmetry are not discussed here, the model presented in this paper can be used as a tool to develop such studies.

The catalytic configurations studied in this paper have been inspired by existing models and ideas in the origin-of-life field (autocatalytic sets, hypercycles, etc), but they only represent a tiny fraction of what can be analyzed with our model. Various extensions (e.g. adding achiral species, including more than one food molecule, including inhibition, considering random networks and rate constants, etc) are left for future research. In particular, having more than one food molecule would allow the possibility of discussing the coding of information (as is accomplished by RNA and DNA).

## Data Availability

The numerical datasets produced and analyzed during the current study are available from the corresponding author upon reasonable request.
